# Local amphotericin B for a neglected *Rhizopus microsporus* necrotizing soft tissue infection following crush syndrome: a case report

**DOI:** 10.3389/fmed.2026.1817919

**Published:** 2026-04-29

**Authors:** Weiwei Yan, Zichao Li, Liming Wang, Jihong Zhang, Peng Chen

**Affiliations:** 1 Intensive Care Unit, Weifang People’s Hospital, Weifang, China; 2 Department of Cardiac Critical Care and Rehabilitation, Weifang People’s Hospital, Weifang, China

**Keywords:** crush syndrome, debridement, mucormycosis, *Rhizopus microsporus*, targeted next-generation sequencing, topical amphotericin B

## Abstract

Cutaneous mucormycosis is a highly fatal infection increasingly seen after severe trauma. We present a previously healthy 45-year-old man who developed an invasive *Rhizopus microsporus* infection following a haystack crush injury. Initially treated for crush syndrome and acute kidney injury (AKI), he developed distinct right temple necrosis on hospital day 13. Immediate debridement exposed bone involvement. Notably, standard serum (1,3)-β-D-glucan and galactomannan tests were non-contributory in this case and did not exclude mucormycosis. However, histopathology and targeted next-generation sequencing (tNGS) confirmed *R. microsporus*. Navigating his AKI, we utilized a renal-sparing approach: systemic amphotericin B cholesteryl sulfate complex (ABCD) combined with topical amphotericin B wound dressings. He later successfully stepped down to oral isavuconazole. At the 3-month post-discharge follow-up, the infection had entirely resolved with excellent granulation. This case highlights a critical clinical trap: standard serum (1,3)-β-D-glucan and galactomannan tests can remain completely negative in mucormycosis. Clinicians must rely on early pathology and tNGS for trauma-induced necrosis. Furthermore, coupling aggressive debridement with combined systemic and topical antifungal therapy may represent a useful management strategy in selected critically ill patients with renal impairment.

## Introduction

Cutaneous and soft-tissue mucormycosis is a rapidly progressive and highly lethal opportunistic infection. It constantly challenges current protocols in clinical infection management and surgical care. The responsible pathogens are environmental molds belonging to the *Mucorales* order. While these fungi naturally thrive in soil and decaying matter, human disease is generally driven by only a few specific genera. *Rhizopus*, for instance, is the most frequently isolated genus in clinical practice and accounts for the vast majority of cases (1).

Historically, clinicians considered soft-tissue mucormycosis a disease strictly limited to severely immunocompromised patients—such as transplant recipients or those with unmanaged hematologic malignancies and diabetic ketoacidosis (1). However, recent global data tell a different story. Direct trauma is actually the most common transmission route today, making up about 54% of confirmed cutaneous mucormycosis cases (2). This usually happens when blast injuries, car accidents, or natural disasters forcefully embed fungal spores deep into the tissues (3–6). When confined to the skin and subcutaneous layers, the mortality rate hovers between 18 and 36%. But the clinical picture deteriorates rapidly if the fungal hyphae breach local blood vessels and disseminate to the lungs, brain, or gut. Once systemic, mortality skyrockets to 70–80%. This massive jump in lethality highlights exactly why we need early recognition, aggressive surgical debridement, and strategies to prevent vascular invasion (2).

Here, we report a case of *Rhizopus microsporus* soft-tissue necrosis following crush syndrome. The infection showed local invasion into muscle and adjacent bone confirmed by pathology. We managed to control it using a combination of systemic and topical amphotericin B, followed by oral isavuconazole. Because early symptoms were so vague, the diagnosis was not made until obvious muscle necrosis appeared. This case serves as a stark reminder that trauma-induced fungal infections demand immediate clinical suspicion.

## Case presentation

A 45-year-old male feed mill worker was admitted in August 2025 following a severe crush injury. Upon assessing his baseline risk factors, he was found to be previously healthy, with no history of diabetes mellitus, hematologic malignancies, or prior immunosuppressive therapies—conditions historically considered prerequisites for soft-tissue mucormycosis. His family history was non-contributory, and he had no notable history of smoking, alcohol abuse, or illicit drug use. Psychosocially, he was an active provider for his family. However, his occupation as a feed mill worker involved chronic environmental exposure to agricultural dust and decaying organic matter, which may have served as a predisposing occupational risk factor. The acute event occurred when he was trapped under a haystack for 2 h. Right after being rescued, he complained of severe pain in the crushed areas—his left limbs and the right side of his face—and soon developed oliguria with dark, soy-sauce-colored urine.

**Figure 1:**
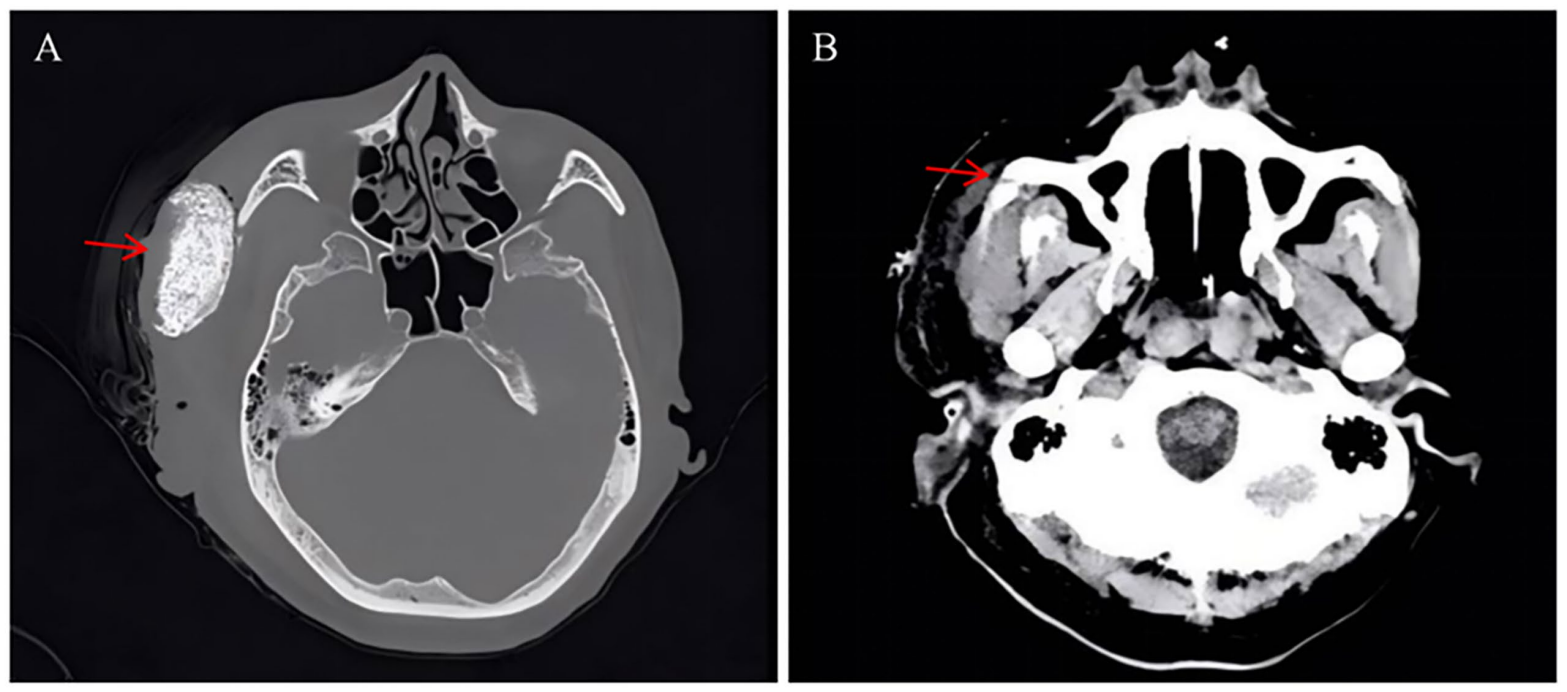
Maxillofacial computed tomography (CT) findings. **(A)** Axial CT image obtained after the initial surgical debridement. The red arrow indicates the extensive tissue defect which has been packed with radiopaque surgical gauze for hemostasis and drainage. **(B)** Axial CT image demonstrating the underlying facial trauma. The red arrow highlights the fracture of the right zygomatic bone, accompanied by significant adjacent soft tissue swelling.

On admission, his hemodynamics and breathing were stable. His left thigh was noticeably swollen, red, tender, and difficult to move. Regarding his initial wound management, he had undergone basic wound irrigation and primary closure in the emergency department for a superficial laceration over his right zygomatic bone. Lab results were alarming: free myoglobin >400 mg/L, creatine kinase 432,613 U/L, LDH 11,143 U/L, AST 5,650 U/L, and creatinine 486 μmol/L. Blood urea nitrogen was 23 mmol/L. Routine serum (1,3)-β-D-glucan and galactomannan assays were negative, with values of <10 pg/mL (reference range: <60 pg/mL) and 0.15 μg/L (reference range: <0.25 μg/L), respectively. The assays were performed using the β-D-glucan detection kit (photometric method; Beijing Jinshanchuan Technology Development Co., Ltd.) and the *Aspergillus* Galactomannan Detection Kit (chemiluminescence assay; Tianjin Xinuo Biomedical Co., Ltd.). A CT scan confirmed the massive left thigh swelling and a right zygomatic fracture (Figure 1B). We diagnosed him with crush syndrome complicated by rhabdomyolysis and acute kidney injury (AKI). He was transferred to the ICU for continuous renal replacement therapy (CRRT), alongside broad-spectrum prophylactic antibiotics (piperacillin sodium and tazobactam sodium), routine sterile wound care and dressing changes for the facial laceration, oxygen, and nutritional support.

**Figure 2:**
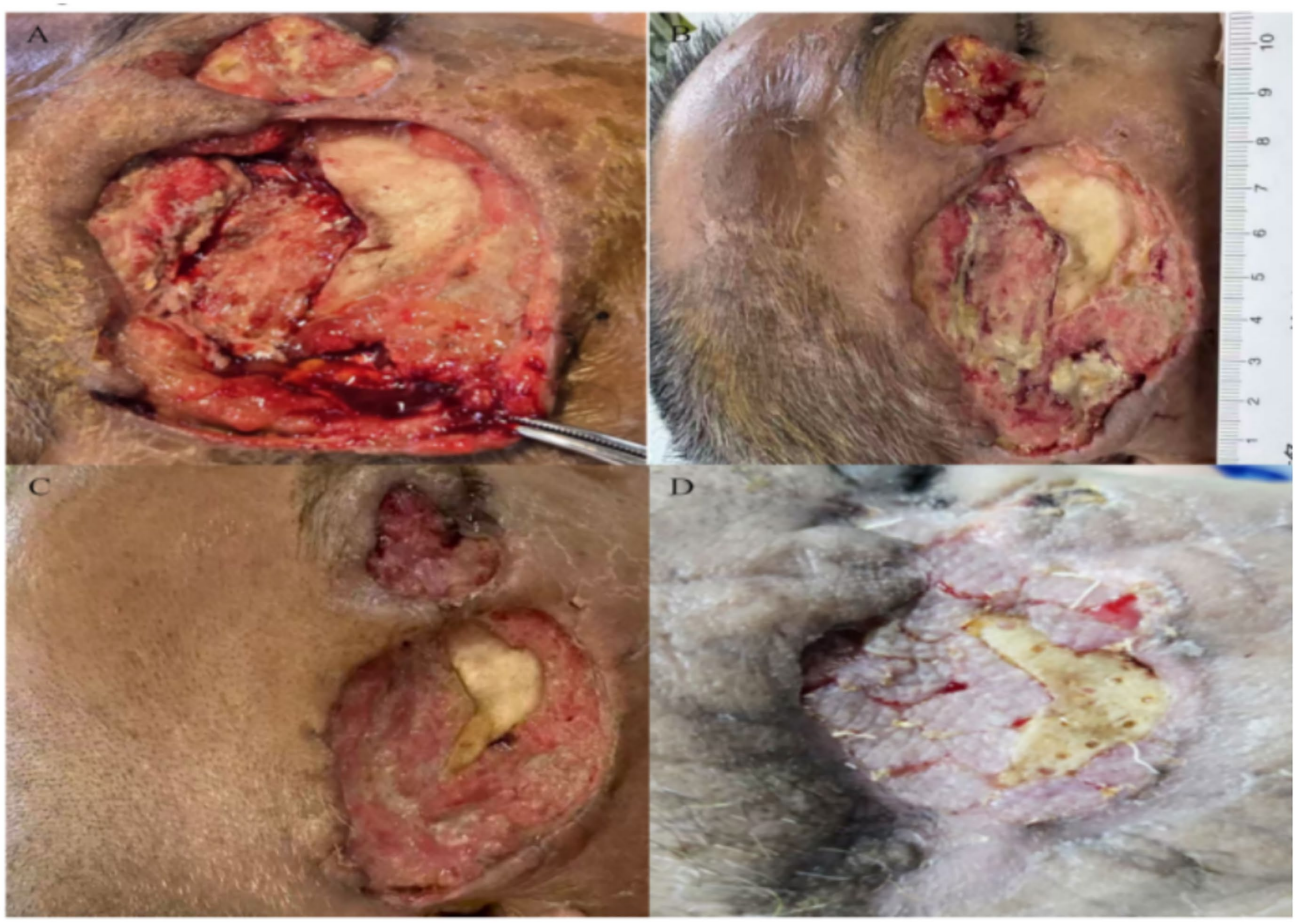
Serial clinical photographs illustrating the progressive healing of the right maxillofacial defect following surgical debridement and targeted antifungal therapy. **(A)** Hospital day 13: The wound bed immediately following the initial aggressive surgical debridement, revealing an extensive tissue defect with exposed zygomatic bone. **(B)** Hospital day 28 (15 days post-debridement): Early formation of healthy granulation tissue at the wound periphery. The adjacent metric ruler was utilized for serial wound measurements. **(C)** Hospital day 43 (30 days post-debridement): Substantial wound contraction with prominent, pink granulation tissue proliferating across the wound base. **(D)** Outpatient follow-up at 3 months post-discharge: The *Rhizopus microsporus* infection is completely resolved. Healthy peripheral granulation tissue has partially covered the previously exposed bone, resulting in a stable and clean wound bed prepared for elective flap reconstruction.

**Figure 3:**
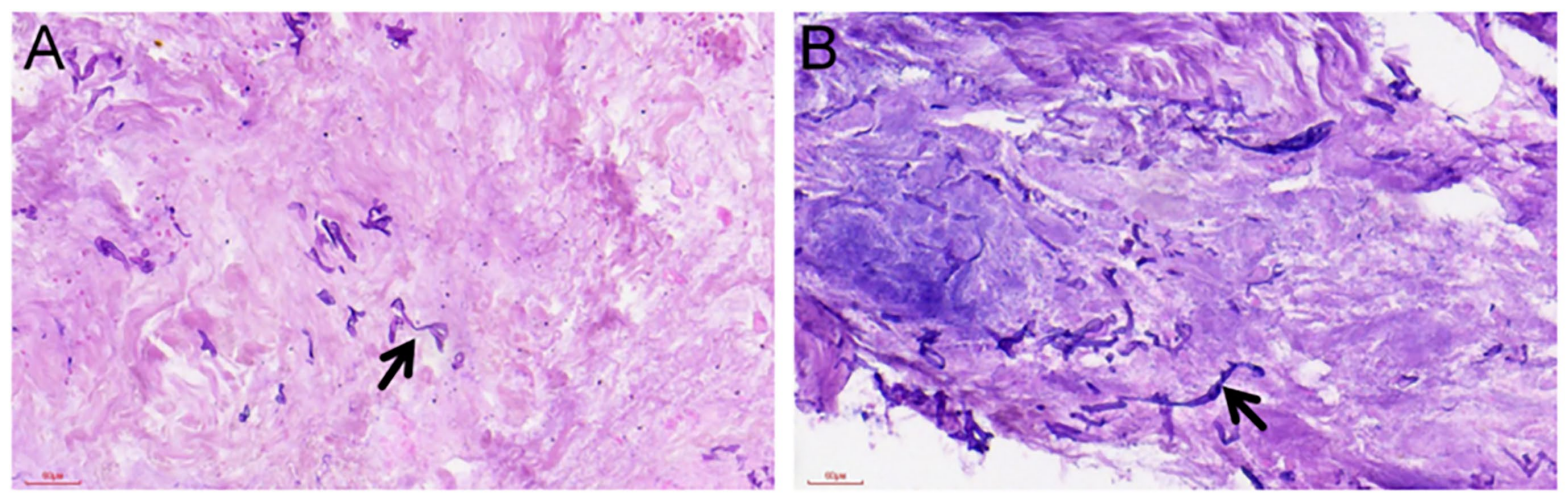
Histopathological examination of the debrided necrotic tissue. **(A)** Tissue section obtained after decalcification of the zygomatic bone. **(B)** Necrotic tissue from the right temporal muscle. Numerous fungal elements are visible within the degenerative and necrotic material (black arrows). The hyphae are broad, non-septate, and of irregular thickness; some cross-sections appear cystic, while others are ribbon-like and folded. This distinctive morphology is highly characteristic of Mucorales invasion. All sections were stained with hematoxylin and eosin (H&E). Scale bars = 60 μm.

By day 13, the leg swelling had improved, but his kidneys had not recovered. At the same time, the crushed area on his right temple turned black. The necrotic patch had distinct borders and no exudate. Maxillofacial surgeons stepped in to debride the wound. During surgery, they found a 7.5 × 4.5 cm area of ulcerated, blackened temporal muscle extending right down to the bone. Removing the dead tissue exposed a necrotic bone fragment underneath (Figure 2). Because the defect was large and close to the eye, primary closure or negative pressure wound therapy was impossible. Instead, we packed the cavity with iodoform gauze and applied a pressure dressing (Figure 1A). During this initial debridement on hospital day 13, samples of the deep necrotic temporal muscle and adjacent bone scrapings were immediately sent for histopathology, traditional fungal culture, and targeted next-generation sequencing (tNGS). Simultaneously, peripheral venous blood was collected for adjunctive tNGS testing. Traditional tissue fungal cultures ultimately yielded no growth, likely due to the characteristic fragility of *Mucorales* hyphae during tissue homogenization. Pathology of the excised tissue, however, revealed numerous fungal hyphae consistent with *Mucorales* (Figure 3). Serum (1,3)-β-D-glucan and galactomannan tests were repeated and both remained completely negative (Glucan: <10 pg/mL; Galactomannan: <0.10 μg/L). However, tNGS (performed using the Illumina NextSeq 550Dx sequencing platform) identified *Rhizopus microsporus* in the tissue specimen and also detected Rhizopus DNA in peripheral blood within 48 h. Environmental or laboratory contamination was considered less likely because the tissue specimen was obtained intraoperatively under sterile conditions, and the tissue tNGS result was concordant with histopathological evidence of *Mucorales* invasion. The peripheral blood tNGS finding was interpreted cautiously as supportive molecular evidence.

These findings supported a clinical diagnosis of locally invasive mucormycosis with deep muscle and adjacent bone involvement. Peripheral blood tNGS positivity indicated circulating fungal DNA but did not establish proven systemic dissemination. Based on the tNGS and pathology results, we initiated systemic antifungal therapy using intravenous amphotericin B cholesteryl sulfate complex (ABCD) at a dose of 5 mg/kg/day. The patient (body weight: 73 kg) tolerated the treatment well alongside continuous renal replacement therapy. Intravenous ABCD was initiated on hospital day 16 and administered for a total duration of 45 days, reaching a cumulative dose of 16,425 mg. As the patient’s renal function recovered and the facial wound exhibited healthy granulation tissue, we planned a step-down to oral therapy. To ensure adequate therapeutic drug levels during the transition, an overlapping strategy was employed. On hospital day 58, oral isavuconazole was introduced with a standard loading dose of 200 mg every 8 h for 48 h. Subsequently, on hospital day 60, intravenous ABCD was permanently discontinued, and the patient was successfully transitioned to a long-term maintenance dose of oral isavuconazole at 200 mg once daily.

For local control, we established a targeted, two-phase topical amphotericin B (AMB) protocol. We utilized amphotericin B for injection (North China Pharmaceutical Co., Ltd., China; 5 mg/vial). Crucially, because AMB precipitates in the presence of saline or electrolytes, all drug reconstitution was performed strictly using sterile 5% dextrose. The topical antifungal solution was freshly prepared immediately prior to each use by dissolving a total absolute dose of 2.5 mg (exactly half a vial) of AMB into 100 mL of 5% dextrose, yielding a final concentration of 0.025 mg/mL (25 mcg/mL). During each dressing change, this entire 100-mL volume was used to irrigate the wound cavity, followed by packing the defect with sterile gauze soaked in the same solution. An opaque outer bandage was applied to protect the drug from light degradation.

To match the clinical progression of the wound, we employed an intensive strategy initially: for the first 25 days, meticulous bedside debridement and this specific AMB dressing change were performed once daily. As the necrotic tissue cleared and healthy granulation emerged, the frequency was stepped down to every other day for the remaining 20 days. In total, this rigorous topical application was administered exactly 35 times. Importantly, this topical protocol was co-administered simultaneously with the systemic intravenous ABCD. Because the necrotic tissue had been entirely cleared and a well-vascularized granulation bed had successfully formed, both the intravenous and topical AMB routes were permanently discontinued together on hospital day 60. From that point forward, the patient was maintained solely on oral isavuconazole.

**Figure 4:**
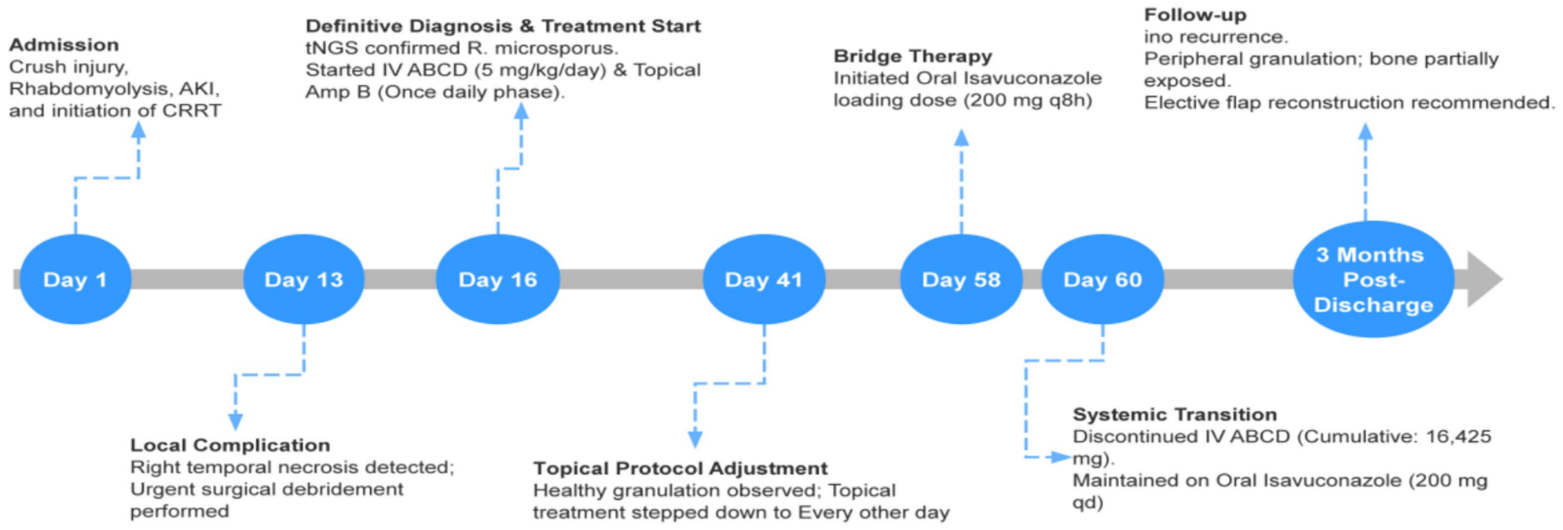
Timeline of the patient’s clinical course and antifungal management. The timeline illustrates key clinical milestones, starting from the initial crush injury to the 3-month post-discharge follow-up. It details the urgent surgical interventions, the definitive diagnosis via tNGS, and the specific systemic and topical antifungal regimens, including cumulative dosages and the transition from intravenous to oral therapy.

Gradually, his renal function recovered alongside the continuous renal replacement therapy. As detailed in the clinical trajectory (Table 1), his serum creatinine progressively dropped from a peak of 524 μmol/L (Day 13) to 217 μmol/L by Day 60, and eventually normalized to 102 μmol/L at the 3-month follow-up. Concurrently, the facial wound exhibited a remarkably favorable response to the combined treatment. By hospital day 28 (15 days post-debridement), early healthy granulation tissue began to emerge at the wound periphery. By day 43 (30 days post-debridement), prominent pink granulation tissue had proliferated across the extensive wound base, effectively replacing the previously necrotic zones and partially covering the exposed zygomatic bone. After nearly 60 days of hospitalization, the patient was successfully switched to oral isavuconazole (200 mg once daily) for long-term maintenance therapy. At the 3-month outpatient follow-up, the infection was deemed completely resolved (Figure 2D). Crucially, the assessment of fungal clearance was established clinically and macroscopically by the trauma surgical team, based on the robust, sustained formation of well-vascularized granulation tissue and the absolute absence of new necrotic eschar or active exudate. A repeat invasive tissue biopsy or follow-up tNGS was not performed; once healthy tissue was achieved, further surgical sampling was strictly avoided to prevent iatrogenic damage to the fragile granulation bed, which had been carefully preserved for the patient’s scheduled elective flap reconstruction. The complete treatment process and key clinical milestones are illustrated in the timeline (Figure 4). To quantitatively illustrate the patient’s clinical trajectory, the serial trends of renal function recovery (serum creatinine and estimated GFR) and the progressive reduction in the facial wound dimensions are summarized in Table 1. These objective metrics demonstrate the efficacy and renal safety of the combined systemic and topical antifungal strategy.

**Table 1:** Serial trends in renal function recovery (serum creatinine and estimated GFR) and reduction in facial wound dimensions during treatment and follow-up.

Clinical milestone (hospital day)	Serum creatinine (μmol/L)	Estimated GFR (mL/min/1.73m^2^)^a^	Wound dimensions (length × width, cm)^b^	Wound bed characteristics
Day 1 (admission)	486	12.24	Intact/contusion	Swelling, red, tender
Day 13 (first debridement)	524	11.18	7.5 × 4.5	Black necrotic eschar, bone exposed
Day 28 (mid-treatment)	428	14.25	6.5 × 3.5	Early granulation
Day 43 (continued Tx)	464	12.93	5.0 × 2.5	Prominent pink granulation tissue
Day 60 (systemic transition)	217	32.20	3.0 × 2.0	Substantial contraction, ready for step-down
3 months post-discharge	102	79.66	2.5 × 1.5	Approaching the healing stage

a eGFR was calculated using the CKD-EPI equation.

b Wound dimensions were estimated based on photographic assessment with reference to the scale marker in Figure 2B.

Regarding treatment-related adverse events, the systemic ABCD was well-tolerated. It did not exacerbate the patient’s acute kidney injury; rather, his renal function steadily recovered during the 45-day intravenous course. Conversely, the primary adverse event was associated with the topical therapy. The patient experienced severe local pain and intense burning sensations during and immediately following the amphotericin B wound irrigation and wet dressing applications. Although physically and emotionally exhausting for the patient, this local toxicity did not result in further tissue necrosis and was managed with appropriate systemic analgesia. Crucially, these local side effects did not require the discontinuation of the life-saving topical protocol.

## Discussion

This case highlights how easily a traumatic spore inoculation can be overlooked until it causes catastrophic tissue death. Initially, the local reaction mimics a minor skin contusion or ordinary bacterial cellulitis. This mimicry is dangerous. As the distinct black necrosis emerged on day 13, our formal differential diagnosis included both non-infectious and infectious etiologies. Non-infectious ischemic pressure necrosis from the initial crush injury was considered; however, the rapid and aggressive tissue destruction extending down to the bone strongly indicated an invasive infectious process. Bacterial necrotizing fasciitis (e.g., caused by *Streptococcus pyogenes*, *Staphylococcus aureus*, or *Pseudomonas aeruginosa*) is common following trauma. Yet, the distinct lack of purulent exudate, combined with the catastrophic progression despite broad-spectrum prophylactic antibiotics, raised a high clinical suspicion for an angioinvasive fungal infection. Among fungal pathogens, Aspergillus, Fusarium, and Mucorales can all present with rapidly progressive necrotic eschars. The persistently negative serum (1,3)-β-D-glucan and galactomannan results were not supportive of biomarker-detectable fungal infections such as aspergillosis in this clinical context, but they did not by themselves exclude other fungal pathogens. By the time we performed the aggressive diagnostic debridement, the fungus had already invaded the subcutis and nearby bone, turning a localized problem into a severe deep-tissue infection (7). Ultimately, histopathology demonstrated *Mucorales* tissue invasion, and tissue tNGS identified *Rhizopus microsporus*. Peripheral blood tNGS also detected Rhizopus DNA, but this molecular finding alone did not establish proven disseminated infection. This pathogen profile differs from the predominance of *Candida* species reported in some Asian trauma-associated fungal infections (2). In the absence of radiologic, pathologic, or microbiologic evidence of extracutaneous organ involvement, the peripheral blood tNGS result was interpreted as supportive molecular evidence rather than proof of systemic spread.

In a healthy host, embedded Mucorales spores are usually controlled by innate host defenses, while intact skin serves as an important barrier against inoculation (7). However, massive trauma can profoundly alter the local tissue environment. Tissue ischemia, necrosis, and possible trauma-associated immune dysregulation may create a permissive setting for spore germination and invasion (8). Once established, Mucorales hyphae aggressively penetrate blood vessel walls. This angioinvasion triggers thrombosis and progressive downstream ischemia, culminating in extensive coagulative necrosis of the surrounding tissues (1). This poorly perfused, devitalized zone may further limit the access of circulating immune cells such as neutrophils and reduce the penetration of systemic antifungal agents into the core of infection (8). These pathophysiological features help explain the need for prompt and aggressive surgical debridement (8). Crush syndrome itself should not be regarded as a classical systemic immunosuppressive condition, and in this case, we do not infer the presence of pre-existing systemic immunodeficiency. Rather, we believe that post-traumatic mucormycosis developed in the setting of direct wound inoculation, extensive soft-tissue destruction, tissue necrosis, and a highly permissive local microenvironment. Altered tissue perfusion, acidosis, and other metabolic derangements associated with crush injury may also have contributed, although these mechanisms were not directly demonstrated in our patient. In addition, major trauma may induce transient systemic immune dysregulation, which could have further impaired host defenses. Therefore, instead of describing crush syndrome as a direct immunosuppressive factor, we consider it a severe clinical context in which profound local tissue injury, contamination, and possible trauma-associated immune dysfunction together facilitated mucormycosis.

Histopathology remains a cornerstone for the diagnosis of invasive mucormycosis, as the demonstration of broad, ribbon-like, pauci-septate hyphae with tissue or vascular invasion provides strong evidence of invasive fungal disease (9). However, histopathology alone cannot reliably determine the causative genus or species. In this context, molecular methods such as qPCR and tNGS serve as important adjunctive tools. They can provide rapid adjunctive molecular identification, although their taxonomic resolution depends on assay design and target coverage (10). Unlike metagenomic next-generation sequencing (mNGS), which sequences all nucleic acids in a specimen in an unbiased manner, tNGS enriches predefined pathogen-specific targets through multiplex PCR before sequencing. This targeted strategy may be particularly advantageous in tissue specimens with abundant host DNA, where fungal nucleic acids may be present at low abundance and therefore be more difficult to detect by mNGS. By reducing host background interference, tNGS may improve analytical sensitivity, shorten turnaround time, and lower cost for pathogens included in the assay panel. However, these advantages are restricted to predefined targets, and tNGS may miss unexpected or non-target organisms. In the present case, tNGS contributed to the rapid identification of *Rhizopus microsporus*, complementing the histopathological findings and supporting the final diagnosis.

A critical diagnostic takeaway for clinicians is the behavior of standard serum biomarkers. In this case, serum (1,3)-β-D-glucan and galactomannan assays were negative. This finding is compatible with mucormycosis, as *Mucorales* are not reliably detected by these assays, which may remain negative despite proven disease (11). In addition, false-positive β-D-glucan results may occur because of exposure to glucan-containing surgical gauze, blood products, or cellulose-based dialysis materials (12, 13), whereas modern synthetic CRRT membranes are less clearly associated with such interference. In our patient, the use of synthetic CRRT membranes rather than cellulose-based filters, together with rigorous sampling procedures, may have reduced potential sources of assay interference. Therefore, this negative biomarker profile should be interpreted as non-contributory rather than diagnostic: it did not rule out invasive mucormycosis, but it remained consistent with the clinicopathological suspicion and underscored the need for prompt tissue-based and molecular confirmation.

The ECMM/ISHAM global guidelines emphasize three pillars of treatment: reverse immunosuppression, perform repeated surgical debridements, and start high-dose intravenous antifungals immediately (14). You should not even wait for final lab confirmation if the clinical picture fits (15). The guidelines strongly recommend liposomal amphotericin B (L-AmB) or newer broad-spectrum triazoles (14). In our case, the patient’s crush-induced AKI complicated drug selection. To preserve the patient’s residual renal function, we selected ABCD, which was considered a renal-sparing option in this clinical setting compared to conventional formulations. We successfully transitioned him to oral isavuconazole for long-term step-down therapy post-discharge. Crucially, we bypassed the ischemic drug-delivery barrier by applying amphotericin B directly to the wound bed. Given the absence of standardized international guidelines for topical AMB dosing, empirically reported concentrations in the literature vary widely, often ranging from 50 to 200 mcg/mL. We deliberately selected a more conservative concentration of 0.025 mg/mL (2.5 mg, or half a 5-mg vial, dissolved in 100 mL of 5% dextrose). This specific dose was meticulously chosen to achieve a potent local fungicidal effect within the avascular necrotic bed while stringently minimizing the risks of severe local tissue irritation and chemical necrosis. Furthermore, this controlled concentration mitigated the risk of unintended systemic drug absorption through the extensive wound bed—a critical safety consideration given the patient’s crush-induced acute kidney injury. This combined systemic-topical approach effectively halted the Rhizopus invasion, allowing the wound to heal enough for an elective flap transplant.

## Patient perspective

During follow-up, the patient described his experience as follows: “When I was first trapped under the haystack, I thought I was going to die. However, being informed that a rare, rapidly invasive fungal infection was progressively destroying my facial soft tissues down to the bone represented my darkest moment. The treatment was physically and emotionally exhausting. Repeated debridement procedures and severe burning sensations induced by topical antifungal dressings were extremely difficult to tolerate. Nevertheless, as weeks passed, I gradually observed the black necrotic tissue being replaced by healthy pink granulation tissue, which restored my hope. I am profoundly grateful to the medical team for saving my life, preserving renal function, and salvaging my facial appearance. I now look forward to reconstructive flap surgery and a full return to normal life.”

## Conclusion

Severe trauma may predispose patients to mucormycosis by creating a hypoxic and ischemic tissue environment and facilitating the direct inoculation of environmental fungi into the wound, particularly in contaminated settings. Serum (1,3)-β-D-glucan and galactomannan tests may be non-contributory in this setting. In this case, early diagnosis using histopathology and tNGS, combined with surgical debridement and both systemic and topical antifungal therapy, was associated with favorable clinical outcomes. This approach may represent a useful management strategy in similar clinical scenarios, although treatment should be individualized and further evidence is needed.

## Data Availability

The original contributions presented in the study are included in the article/supplementary material, further inquiries can be directed to the corresponding author.
